# Ipsilateral Uveitis and Optic Neuritis in Multiple Sclerosis

**DOI:** 10.1155/2012/372361

**Published:** 2012-11-19

**Authors:** Eric Thouvenot, Frédéric Mura, Marie De Verdal, Bertrand Carlander, Mahmoud Charif, Christelle Schneider, Sophie Navarre, William Camu

**Affiliations:** ^1^Neurology Department, Hôpital Carémeau, 9 Place Robert Debré, 30029 Nimes Cedex 9, France; ^2^Ophthalmology Department, Hôpital Gui de Chauliac, 34295 Montpellier Cedex 5, France; ^3^MS Clinic, Neurology Department, Hôpital Gui de Chauliac, 34295 Montpellier Cedex 5, France

## Abstract

*Background*. Uveitis is 20 times more frequent in multiple sclerosis (MS) patients than in the general population. *Methods*. A retrospective study of local multiple sclerosis (*n* = 700) and uveitis cohorts (*n* = 450) described the ophthalmological and neurological characteristics of patients with multiple sclerosis and uveitis. *Results*. Uveitis and multiple sclerosis were associated in seven patients. The time intervals between diagnoses of MS and uveitis ranged from 6 months to 15 years. Analysis of the patients' characteristics revealed that multiple sclerosis was associated with an older age of onset than usually expected, that is, 39 years. Uveitis was bilateral in three cases and mainly posterior (5/10). Five patients presented with acute optic neuritis (two in one eye and three in both eyes). All eyes presenting with acute optic neuritis were also affected by uveitis (*P* = 0.02), though not simultaneously. *Conclusion*. The ipsilateral association between optic neuritis and uveitis in this series of patients with multiple sclerosis may suggest a reciprocal potentiation between optic neuritis and uveitis in multiple sclerosis.

## 1. Introduction

Optic neuritis (ON) is the most frequent ophthalmic manifestation of multiple sclerosis (MS), occurring in ~30% of cases. In contrast, uveitis is only associated with MS in 1% of patients [1]. Similarly, uveitis is attributed to MS in 1%–3% cases of uveitis and is associated with an increased risk of severe visual disability [2, 3]. The clinical features of uveitis in MS vary considerably between reports but generally manifest as bilateral, intermediate (pars planitis) uveitis [1, 4–6]. In a series of 1855 patients with chronic anterior uveitis, MS was diagnosed in 30 patients (1.6%) [7]. Patients with MS and uveitis also present with ON more frequently than expected in MS [4, 5], but a direct pathogenic link between ON and uveitis has not yet been considered. The aim of this study was to determine the prevalence of uveitis in a cohort of patients with MS and to analyze its relationship with ON with regards to both the ophthalmological and neurological characteristics of these patients. 

## 2. Patients and Methods

Patients from Montpellier Hospital with MS-associated uveitis were retrospectively identified through the MS clinic database in the Neurology Department (700 patients) and the uveitis database from the Ophthalmology Department (450 patients). Diagnosis of MS was assessed from clinical and MRI data according to the 2005 revised McDonald criteria for relapsing-remitting or primary progressive MS [8]. Acute uveitis was defined by the observation of acute uveitis or a report of previous acute uveitis with the presence of sequelae. Uveitis was classified according to the SUN classification [9]. Uveitis differential diagnosis was ruled out after comprehensive tests: that is, chest computed tomography, levels of angiotensin-converting enzyme and antinuclear antibodies, antiphospholipid antibodies, Borrelia, Rickettsia, Brucella, human immunodeficiency virus, hepatitis B and C, and toxoplasmosis and syphilis tests. ON was defined by an acute loss of visual acuity or scotoma associated with extended visual evoked potential latencies (>120 ms) and/or an MRI T2 hypersignal of the optic nerve. Bilateral ON and bilateral uveitis corresponded to acute episodes that affected both eyes simultaneously.

Patients identified with MS and uveitis were reassessed in order to confirm their characteristics regarding gender, duration and course of MS, disability caused by MS as assessed by the Expanded Disability Status Scale (EDSS) [10], onset time of uveitis and its type, course and symptoms, the presence of pars planitis or periphlebitis, and whether ON occurred in the left or right eye. Statistical analyses were performed using R software.

## 3. Results

Uveitis was identified in 5 of the 700 patients from the MS clinic (0.7%) (Table 1, patients 1–5), and MS was found in 2 patients from the uveitis database (patients 6 and 7). There were six women and one man. Patient 1 had antecedent thyroiditis, and patient 2 had recovered from hepatitis B virus. Of the seven cases, three had experienced uveitis before the onset of MS (patients 2, 4, and 7), and one patient had recurrent episodes until 3 years after the onset of MS (patient 2). There were no simultaneous manifestations of MS and uveitis. The time intervals between diagnoses ranged from 6 months to 15 years (Table 1).

Among the seven patients with MS and uveitis, one presented with primary progressive MS and six had a relapsing-remitting course (86%). In one case (Patient 1), uveitis appeared during the secondary progressive phase of MS. Mean age of onset of MS in patients with uveitis was older compared to our cohort of MS patients with no uveitis (39 years; range: 17–52 versus 31 years, resp., *P* = 0.14, Wilcoxon rank sum test), although this was not significant. Moreover, 4/7 patients had onset of MS when aged >45 years. EDSS scores ranged from 2 to 6 (median 4). None of the patients had a relapse of MS and uveitis at the same time. MRIs showed classical demyelinating lesions of the brain, fulfilling the Barkhof criteria for dissemination of space, for all patients. The cerebrospinal fluid disclosed oligoclonal bands in three of the four patients tested (patients 3, 5, and 6). Patients received different disease-modifying drugs, but not at the time of the study. Treatments used comprised intramuscular IFNb1a (patients 2, 5, and 6), azathioprine (patients 3, 6, 7), and methotrexate (patient 6).

**Figure 1:**
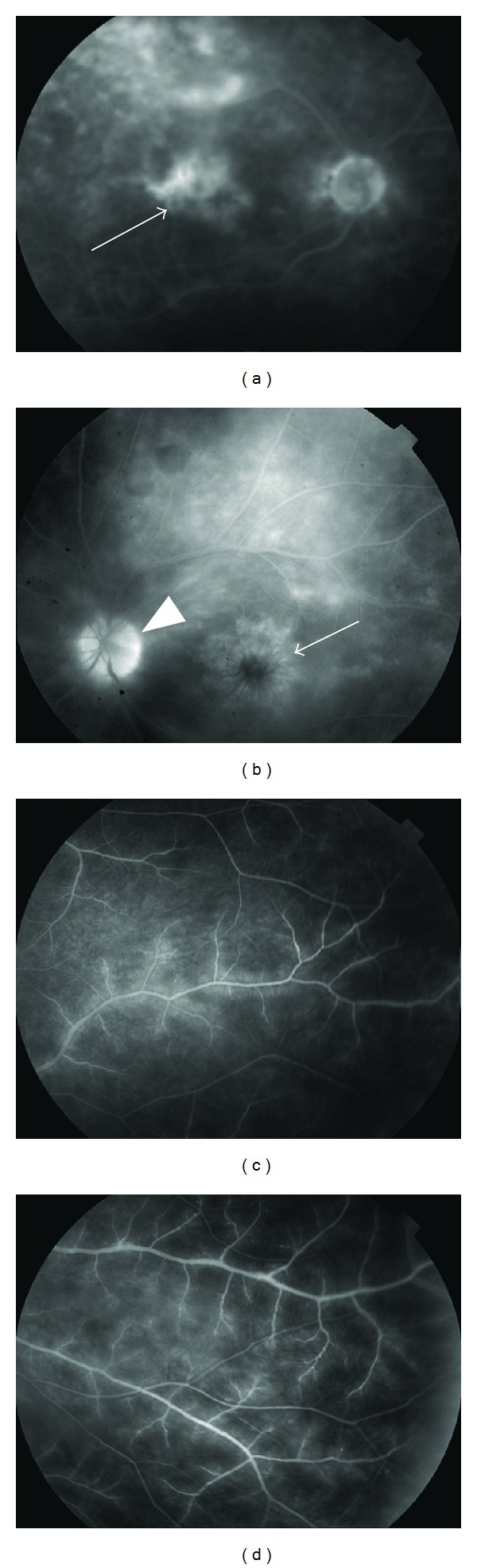
Fundus photographs. Top, late-phase fluorescein angiograms from patient 6 (panuveitis, (a)) showing retinal vasculitis with cystoid macular edema (arrow) and disc leakage (arrowhead), and from patient 7 (posterior uveitis, (b)) showing retinal vasculitis with diffuse cystoid macular edema (arrow). However, because of the severity of the vitritis, the vessels are poorly visualized (a). Bottom, fluorescein angiograms from patient 5 (posterior uveitis, (c) and (d)) showing retinal occlusive vasculitis with arteriolar leakage at a late phase.

Uveitis was observed in 10 eyes (bilateral in three cases) and was of various types: anterior and intermediate (pars planitis) (*n* = 2), anterior (*n* = 1), panuveitis (*n* = 2, bilateral), and posterior (*n* = 5). Disc edema and vasculitis were observed in patients 4–7, macular edema in patients 5–7 (Figure 1), and periphlebitis in only one patient (patient 5). The mean age of onset of uveitis was 40 years (range: 19–62). 

One patient had antecedent acute optic neuritis before uveitis, and four patients presented with acute optic neuritis (bilateral in three cases) after uveitis. All eyes presenting with ON were also affected by uveitis (*P* = 0.02, Fisher's test). All patients with bilateral ON also had bilateral uveitis, and those cases with unilateral ON also had uveitis in the same eye, though not at the same time.

## 4. Discussion

We have described the clinical characteristics of seven patients affected by both uveitis and MS. Uveitis was identified in 0.7% patients from the MS database, a similar proportion observed by other authors [1]. In accordance with a previous report [1], uveitis was mainly posterior (5/10 eyes) whereas MS has been generally associated with intermediate uveitis (pars planitis) [11, 12]. This overrepresentation of posterior uveitis might be caused by the retrospective inclusion of symptomatic patients, without systematic detection of MS patients with asymptomatic pars planitis, as often happens. The frequency of periphlebitis observed by Haarr in a series of 303 patients with multiple sclerosis (22%) is greater than that in our series (only one case, 14%), but we only took into account patients with MS-associated uveitis [13]. As already reported, uveitis was often bilateral [4, 5], and the mean age of uveitis onset was 40 years old [2].

Analysis of patients' characteristics showed two interesting patterns. First, MS onset was late (39 versus 29 years for relapsing-remitting MS in the literature [14]) despite only one patient with primary progressive MS (14%). This difference was not significantly different from our MS cohort (39 versus 31 years), probably due to the small size of our group. Second, for all five patients with ON and uveitis, both inflammatory ophthalmological manifestations always occurred in the same eye. In the literature, acute ON occurs in 25% [5] to 54% [4] of MS patients with uveitis. However, to the best of our knowledge, the occurrence of ON and uveitis in the same eye has not yet been reported. Moreover, analyzing the uveitis database, we could not identify any other cases of ON-associated uveitis (i.e., without MS), suggesting a specific association between uveitis and ON in MS. This is also supported by the higher proportion of ON in patients with MS and uveitis compared to the incidence of ON in MS without uveitis. Moreover, bilateral ON was also more frequent (3/7) in our series than expected in MS without uveitis. Simultaneous bilateral optic neuritis is usually not associated with MS [15]. As bilateral ON was always associated with bilateral uveitis, we hypothesized that uveitis and ON could reciprocally potentiate each other in MS patients.

Uveitis and MS share common genetic risk factors and autoantigens [6, 12, 16]. Both diseases also involve the same type of immune response (autoreactive T cells) [17]. Experimental autoimmune encephalomyelitis, using an injection of S100B (a glial cytosolic protein involved in astrocyte proliferation, neuron survival, and neurite outgrowth in the brain [18]), can induce inflammatory lesions of the cerebral white matter accompanied by panuveitis and retinitis [17]. In another animal model, myelin basic protein-specific T helper cells induced experimental anterior uveitis [19].

## 5. Conclusion

The association of MS and uveitis is well described. However, we report the first observation of a significant association of ipsilateral optic neuritis and uveitis in a series of patients with MS. This suggests a continuum between ophthalmologic inflammatory processes rather than an association between different autoimmune diseases. We propose that this association should be investigated in other series of patients with MS and uveitis.

## Figures and Tables

**Table 1:** Patients' characteristics.

Patient	Gender	Age (years)	Age at MS onset (years)	MS course	Age at uveitis onset (years)	Uveitis evolution	Type of uveitis	Side of uveitis	Occurrence of ON	Side of ON
1	M	66	47	SP	62	Acute	Anterior	L	yes	L
2	F	61	51	RR	49	Chronic	Anterior and intermediate	L	no	—
3	F	57	52	RR	52	Acute	Anterior and intermediate	L	yes	L
4	F	27	24	PP	20	Acute	Posterior	L	no	—
5	F	22	17	RR	19	Acute	Posterior	B	yes	B
6	F	54	49	RR	49	Acute	Panuveitis	B	yes	B
7	F	43	30	RR	28	Acute	Posterior	B	yes	B

Whole group	1M/6F	47	39	5RR/2P	40			10 U		8 ON

M: male; F: female; U: uveitis; ON: optic neuritis; RR: relapsing-remitting MS; P: progressive; SP: secondary-progressive MS; PP: primary progressive MS; L: left; B: bilateral.
